# Reliability of ITV approach to varying treatment fraction time: a retrospective analysis based on 2D cine MR images

**DOI:** 10.1186/s13014-020-01530-6

**Published:** 2020-06-12

**Authors:** Davide Cusumano, Jennifer Dhont, Luca Boldrini, Giuditta Chiloiro, Angela Romano, Claudio Votta, Silvia Longo, Lorenzo Placidi, Luigi Azario, Marco De Spirito, Dirk Verellen, Vincenzo Valentini

**Affiliations:** 1 Fondazione Policlinico Universitario Agostino Gemelli IRCCS, Largo Agostino Gemelli,8, 00168 Rome, Italia; 2 Department of Electronics and Informatics (ETRO), Vrije Universiteit Brussel (VUB), Pleinlaan 9, B-1050 Brussels, Imec, Leuven, Belgium; 3 Faculty of Medicine and Pharmacy, Vrije Universiteit Brussel (VUB), Brussels, Belgium; 4 Department of Radiotherapy, Iridium Kankernetwerk, University of Antwerp (Faculty of Medicine and Health Sciences), Antwerp, Belgium

**Keywords:** Intra-fraction motion management, Internal target volume, MR-guided radiotherapy

## Abstract

**Background:**

Internal Target Volume (ITV) is one of the most common strategies to passively manage tumour motion in Radiotherapy (RT).

The reliability of this approach is based on the assumption that the tumour motion estimated during pre-treatment 4D Computed Tomography (CT) acquisition is representative of the motion during the whole RT treatment. With the introduction of Magnetic Resonance-guided RT (MRgRT), it has become possible to monitor tumour motion during the treatment and verify this assumption.

Aim of this study was to investigate the reliability of the ITV approach with respect to the treatment fraction time (TFT) in abdominal and thoracic lesions.

**Methods:**

A total of 12 thoracic and 15 abdominal lesions was analysed. Before treatment, a 10-phase 4DCT was acquired and ITV margins were estimated considering the envelope of the lesion contoured on the different 4DCT phases.

All patients underwent MRgRT treatment in free-breathing, monitoring the tumour position on a sagittal plane with 4 frames per second (sec). ITV margins were projected on the tumour trajectory and the percentage of treatment time in which the tumour was inside the ITV (%TT) was measured to varying of TFT.

The ITV approach was considered *moderately reliable* when %TT ≥ 90% and *strongly reliable* when %TT ≥ 95%. Additional ITV margins required to achieve %TT ≥ 95% were also calculated.

**Results:**

In the analysed cohort of patients, ITV strategy can be considered *strongly reliable* only for lung lesions with TFT ≤ 7 min (min). The ITV strategy can be considered only *moderately reliable* for abdominal lesions, and additional margins are required to obtain %TT ≥ 95%.

Considering a TFT ≤ 4 min, additional margins of 2 mm in cranio-caudal (CC) and 1 mm in antero-posterior (AP) are suggested for pancreatic lesions, 3 mm in CC and 2 mm in AP for renal and liver ones.

**Conclusions:**

On the basis of the analysed cases, the ITV approach appears to be reliable in the thorax, while it results more challenging in the abdomen, due to the higher uncertainty in ITV definition and to the observed larger intra and inter-fraction motion variability. The addition of extra margins based on the TFT may represent a valid tool to compensate such limitations.

## Background

In the context of Stereotactic Body Radiotherapy (SBRT), the quantification of tumour motion and its correct management during radiotherapy (RT) delivery represent two crucial aspects still under investigation [1, 2].

Studies conducted in the thorax and abdomen demonstrated that the tumour motion due to patient basal breathing can vary up to a few centimetres during a single RT treatment fraction, especially in cranio-caudal (CC) and antero-posterior (AP) directions [3, 4].

Beyond the respiratory-induced motion, other effects can also modify the intra- and inter-fraction tumour position, such as baseline shifts (i.e. sudden changes in the median tumour position) or baseline drifts (i.e. overall slow tumour position change over the course of a treatment fraction) [5–7].

Due to these sources of variability, a sub-optimal management of the tumour motion can introduce significant dosimetric differences between the planned and delivered dose distribution [8–10] .

Active and passive motion management strategies have been developed to compensate tumour motion both in photon and proton radiotherapy. Although the active techniques, such as real-time tumour tracking or gating, are steadily becoming more prevalent in SBRT, the use of passive techniques is still widespread, as it does not require dedicated treatment machines or additional systems to monitor the patient’s breathing [11, 12].

Internal Target Volume (ITV) estimated from 4DCT is to date the most common passive motion management technique. It consists in defining a therapy volume equal to the envelope of the lesion delineated on all phases of the 4DCT and considering this volume in the Planning Target Volume (PTV) determination [13].

In 4D radiation therapy (4DRT) it is common practice to acquire a 4DCT of the patient in free-breathing, dividing the breathing cycle in 10–16 phases for ITV definition and using the average reconstruction for treatment planning and dose calculation [14–17].

As the ITV definition process is often time-consuming, direct delineating of the ITV on the Maximum Intensity Projection (MIP) image was also investigated, but led only to good results in terms of definition accuracy for lung lesions located away from the diaphragm [18].

The reliability of the ITV approach is based on the assumption that the tumour motion estimated during 4DCT acquisition is representative of the one that will take place during the delivery of all the RT fractions.

This assumption has been analysed by the scientific community and often contradicted.

Furthermore, the 4DCT does not include any information related to the variations in the breathing cycle that may occur inter- or intra-fraction, which may depend on the treatment fraction time (TFT) [19, 20].

Apart from creating an artificial motion path of the tumour (a reconstruction representing a so-called movie-loop), the binning of CT-images based on phase or amplitude introduces an additional uncertainty in the ITV determination [21].

Furthermore, sometimes the low soft tissue contrast offered by 4DCT in the abdomen, combined with motion artefacts often present in the reconstructed images, may lead to an incorrect interpretation of the tumour motion, introducing a systematic error in the ITV definition [19, 22].

The recent introduction of hybrid RT systems equipped with on-board magnetic resonance (MR) scanners introduced the possibility to monitor the tumour motion during the entire RT treatment by means of sagittal MR images acquired in cine mode with 4–8 frames per second [23–25].

Although these systems offer a real-time motion monitoring only in the cranio-caudal (CC) and antero-posterior (AP) directions, the analysis of the cine MR images may lead to useful considerations in clinical practice, as already demonstrated by some published experiences [22, 26, 27].

The primary aim of this study was to investigate the reliability of the ITV approach with respect to the TFT, using the cine MR data acquired during MR-guided radiotherapy treatment (MRgRT) of patients affected by lesions located in the abdominal and thoracic regions.

The effective tumour trajectory over the entire RT treatment was extracted, allowing to calculate the percentage of treatment time in which the lesion was within the ITV defined based on the pre-treatment 4DCT acquisition.

TFT today represents a crucial point of the MR-Linac delivery technology: although several improvements are currently under development for low and high Tesla (T) systems, the delivery time required for an MRgRT treatment ranges from 5 to 25 min, considerably longer compared to a standard treatment [28, 29].

.Recent technological developments in the field of non-hybrid RT, also aim at mitigating TFT by minimizing the intra-fraction motion by delivering complex treatment plans at high speed, reducing TFT to a few minutes only [30, 31].

For these reasons, secondary aim of this study was to estimate the maximum TFT that should not to be exceeded to ensure a reliable treatment using the ITV strategy.

## Methods

### Clinical data and treatment workflow

This retrospective study was focused on the analysis of lesions located in the thorax and abdomen, enrolling a total of 27 patients (12 thoracic and 15 abdominal lesions).

All patients received MRgRT treatment on a low-T hybrid system (ViewRay MRIdian, Mountain View, California, USA) that joins a 0.35 T on-board MR scanner with 3 Cobalt-60 sources [32].

A 4DCT was acquired for each patient using a helical CT scanner (HiSpeed DX/i Spiral, General Electrics, Fairfield, Connecticut, USA) with 2.5 mm slice thickness and 1.25 mm in-plane image resolution during treatment simulation. No intravenous contrast agent was administered, according to our institutional standards of procedure. Phase-based reconstruction was performed using an infrared-based Real-time Position Management system (Varian, Palo Alto, California, USA), dividing the breathing cycle of the patient in 10 phases.

All the MRgRT treatments were administered in free breathing, monitoring the tumour position during the whole therapy time acquiring MR images in cine modality and ensuring the accuracy of the dose delivery using a gating strategy.

Cine MR imaging consists of the acquisition of one user defined sagittal plane of 5–7 mm thickness using a true fast imaging with steady state precession (TrueFisp) sequence with a spatial resolution of 3 × 3 mm^2^ and a temporal resolution of 4 frames/second [33].

Before the start of each treatment fraction, the accuracy of the tumour’s contour in the sagittal MR plane chosen for on-line imaging and its correct propagation in the different cine MR frames was verified by a radiation oncologist on a 30 s preview MR acquisition.

At the end of each treatment fraction, tumour motion on treatment cine MR frames was extracted using the tracking-learning-detection (TLD) algorithm optimised for the application on cine TrueFisp MR images, that provides sub-pixel tracking accuracy and precision higher than 95% in motion estimation [34].

.

**Fig. 1:**
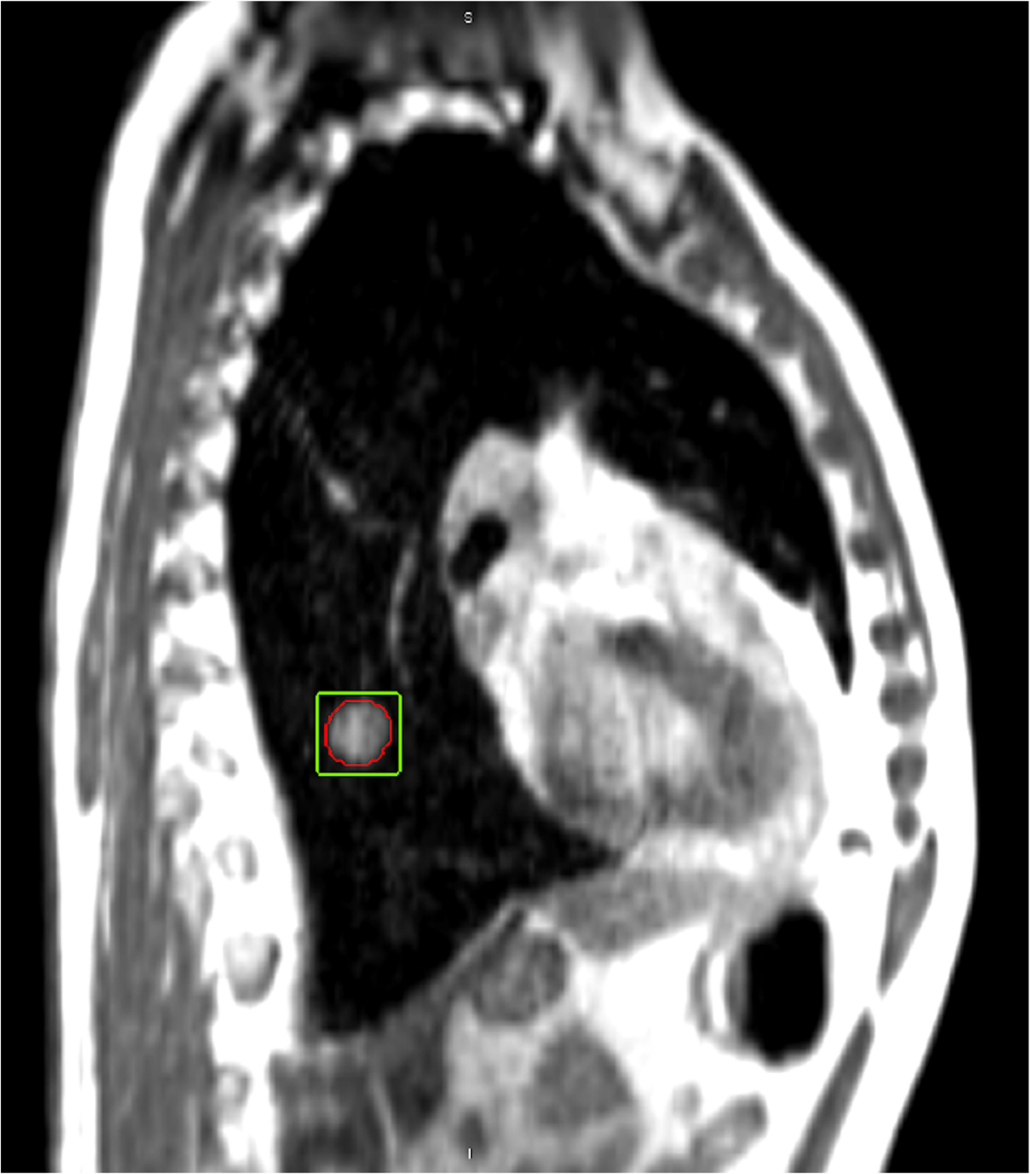
Example of a cine MR frame acquired during the treatment delivery. In red the lesion as delineated by the radiation oncologist and propagated by the software on the MR frame, in green the region of interest defined by the TLD for the extraction of the motion trajectory

Figure 1 shows an example of a cine MR image acquired during treatment delivery.

### Tumour motion analysis

For the ITV definition during 4DCT simulation, the target volume was initially delineated by a radiation oncologist in the 4DCT breathing phase in which the lesion was most visible, and the contours were then propagated with manual corrections to all other breathing phases, according to our standard practice.

Consequently, the ITV was created as the envelope of all these contours obtained in different breathing phases of the 4DCT acquired during simulation.

The ITV was then compared with the effective tumour motion observed during the treatment fractions, extracted from the cine MR images using the TLD algorithm [34].

In particular, for each treatment fraction, the ITV centre was aligned to the mean treatment motion obtained in the first minute of analysis, simulating image-guided patient positioning. Subsequently, the percentage of treatment time in which the tumour was inside the ITV (%TT) was calculated. Deviations inferior to 1 mm were considered negligible for the determination of the %TT.

Five fractions were analysed per patient and the mean %TT was reported with respect to the variation of the TFT from 1 to 10 min (min), in steps of 1 min.

The %TT value after 1 min of treatment was considered as key parameter to analyse the accuracy of the 4DCT in estimating the ITV margins, assuming as negligible the intra-fraction variability in the first minute of treatment.

In the analysis of the %TT with respect to the TFT, the ITV approach was considered *moderately reliable* when %TT was ≥90% and *strongly reliable* when %TT was ≥95%.

The additional ITV margin required to obtain %TT ≥ 95% for all cases where the ITV strategy was not strongly reliable was also calculated.

The correlation between the initial ITV margins estimated in 4DCT and the %TT values obtained in function of the TFT was also investigated calculating the Spearman’s rank correlation coefficient [35]. Correlation was considered significant when the absolute value of the Spearman coefficient |R| was higher or equal to 0.7.

The whole data analysis was performed separately for the thoracic and the abdominal lesions, through in-house scripts developed in R [36].

## Results

**Fig. 2:**
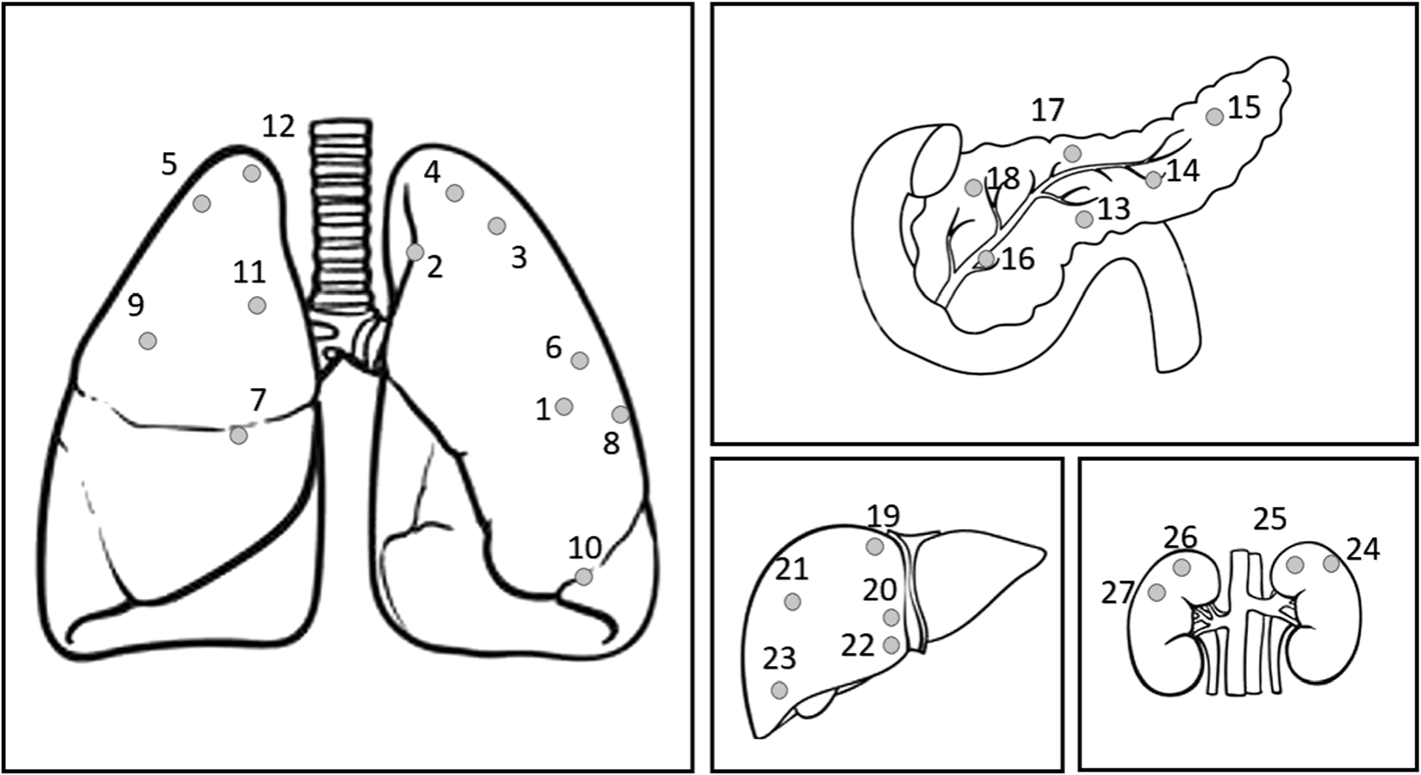
Location observed on 4DCT imaging for the lesions located in the thoracic and abdominal regions

Figure 2 shows the location of the lesions analysed in this study, Table 1 reports the corresponding ITV margins estimated on 4DCT and the mean treatment fraction time for each case, with the corresponding standard deviation.

**Table 1:** ITV margins estimated in CC and AP direction for the analysed lesions located in thoracic and abdominal region as shown in Fig. 1. Mean treatment fraction time and relative standard deviations are also reported for each analysed case

Lesion	Site	CC (mm)	AP (mm)	Mean TFT (min)	Standard Deviation TFT (min)
1	Lung	8	8	10,79	1,32
2	Lung	2	2	10,12	0,89
3	Lung	3	2	9,12	1,16
4	Lung	2	3	12,40	0,77
5	Lung	1	1	9,17	0,65
6	Lung	4	2	12,33	1,11
7	Lung	5	2	9,32	0,90
8	Lung	1	1	10,33	0,58
9	Lung	4	3	9,80	1,01
10	Lung	13	6	10,45	0,87
11	Lung	6	2	12,16	0,92
12	Lung	2	1	9,62	1,19
13	Pancreas	3	3	9,21	0,58
14	Pancreas	5	5	13,45	0,96
15	Pancreas	2	2	13,01	1,58
16	Pancreas	6	3	9,73	1,06
17	Pancreas	4	4	12,46	1,02
18	Pancreas	3	2	8,41	0,82
19	Liver	3	2	9,47	0,75
20	Liver	6	3	10,87	1,68
21	Liver	8	3	8,52	0,92
22	Liver	6	5	11,85	1,77
23	Liver	5	2	8,44	1,40
24	Kidney	7	2	9,05	1,05
25	Kidney	5	2	8,17	0,44
26	Kidney	7	4	9,12	0,80
27	Kidney	6	3	12,16	1,60

The apical lung lesions (2,3,4,5,12) showed an ITV extension ≤3 mm in both the considered directions. The central lung lesions (1,6,7,8,9,11) showed higher motion amplitudes, with ITV margins ranging from 1 to 8 mm. Lesion 8 did not move as it was fixed to the thoracic wall, so its ITV extension was equal to 1 mm in both the considered directions.

The largest ITV margin was observed for lesion 10 (13 mm in CC direction), located in proximity of the diaphragm. The abdominal lesions were located in pancreas (6), liver (5) and kidneys (4).

For these lesions, the predominant motion was observed in CC direction, with a range of 2–8 mm.

The lesions located in liver and kidneys showed higher range in motion amplitude compared to those located in pancreas.

**Fig. 3:**
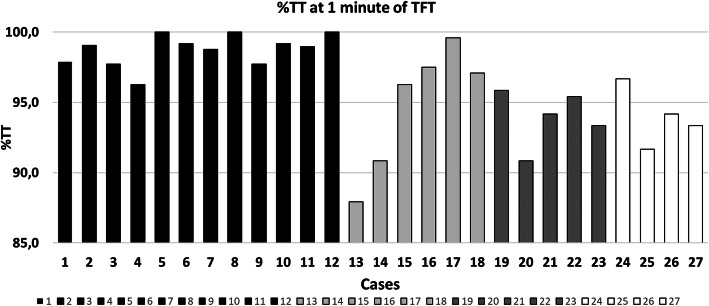
Percentage of TT where the lesion is included in the ITV margin supposing that each treatment fraction would be completed within the first minute of treatment. In black the lung cases, in light grey the pancreatic lesions, in dark grey the liver lesions and in white the kidney lesions

Figure 3 shows the %TT values observed for all the cases, supposing that each analysed treatment fraction would be completed in the first minute of treatment.

The ITV margins estimated on 4DCT ensure %TT > 95% for all investigated lung lesions.

To the contrary, 4DCT accuracy was inferior in case of abdominal lesions, where in 7 out of 14 cases the ITV margin evaluated on 4DCT under-estimated the actual tumour motion, leading to %TT values lower than 95%.

Table 2 reports the mean values of %TT and their relative standard deviations in function of the TFT for all four organs considered in the study.

**Table 2:** Mean values and relative standard deviations of %TT values calculated with respect to the TFT variation for lesions located in lung, pancreas, liver and kidney

TFT (min)	%TT			
	Lung	Pancreas	Liver	Kidney
1	98,7 ± 1,1	94,9 ± 4,5	93,9 ± 2,0	94,0 ± 2,1
2	98,2 ± 1,7	93,8 ± 5,0	92,0 ± 3,9	92,9 ± 1,4
3	98,1 ± 1,5	93,2 ± 4,7	90,9 ± 4,1	92,3 ± 1,9
4	98,0 ± 1,5	93,1 ± 4,4	89,6 ± 3,2	90,6 ± 3,4
5	97,0 ± 2,8	93,2 ± 4,0	88,8 ± 2,8	90,8 ± 2,7
6	96,1 ± 4,2	92,3 ± 4,9	88,1 ± 1,7	90,4 ± 2,5
7	95,3 ± 5,6	91,9 ± 4,2	87,3 ± 1,8	90,3 ± 2,3
8	94,7 ± 6,6	91,6 ± 4,1	86,5 ± 2,8	89,6 ± 2,4
9	94,1 ± 7,7	90,8 ± 3,9	85,6 ± 3,7	88,1 ± 3,4
10	92,5 ± 10,2	90,4 ± 4,2	85,3 ± 4,1	86,8 ± 4,7

For all the considered sites, the %TT decreases with increasing TFT: values higher than 95% were observed only in case of lung lesions for TFT ≤ 7 min.

Table 3 reports the results obtained for the Spearman’s correlation analysis, used to investigate the relationship between the %TT and ITV margins estimated on 4DCT to varying of TFT.

**Table 3:** Spearman’s correlation coefficient calculated to investigate the relationship between the %TT and ITV margins estimated on 4DCT. Correlation was considered significant when |R| ≥ 0.7

TFT	Lung		Liver		Pancreas	
	CC	AP	CC	AP	CC	AP
1 min	−0,23	−0,59	−0,36	0,41	0,35	0,00
2 min	−0,17	−0,43	−0,05	−0,14	0,26	0,00
3 min	−0,22	−0,45	−0,05	− 0,14	− 0,03	− 0,12
4 min	− 0,42	−0,52	− 0,05	−0,14	0,12	0,15
5 min	**−0,70**	−0,59	− 0,21	−0,55	0,12	0,15
6 min	**−0,71**	−0,54	− 0,67	−0,64	− 0,08	−0,14
7 min	**−0,74**	− 0,50	−0,67	− 0,55	−0,32	− 0,46
8 min	**− 0,77**	−0,41	− 0,67	−0,64	− 0,60	**−0,99**
9 min	**−0,79**	− 0,48			−0,48	**− 0,86**
10 min	**− 0,91**	−0,59			**−0,72**	**-0,74**

The analysis was not carried out for kidney lesions due to the low number of cases available (four).

No significant correlation was observed for any lesion site when TFT < 5 min.

In general, the correlation analysis showed that for TFT ≥ 5 min the %TT decreases much more in patients with larger motion estimated in 4DCT.

A significant correlation (|R| ≥ 0.7) was observed in case of pancreatic lesions for AP direction with TFT > 7 min and in the case of lung volumes for CC direction with TFT ≥ 5 min.

A value of R = − 0.67 was observed for liver in CC direction, when TFT ≥ 6 min.

**Fig. 4:**
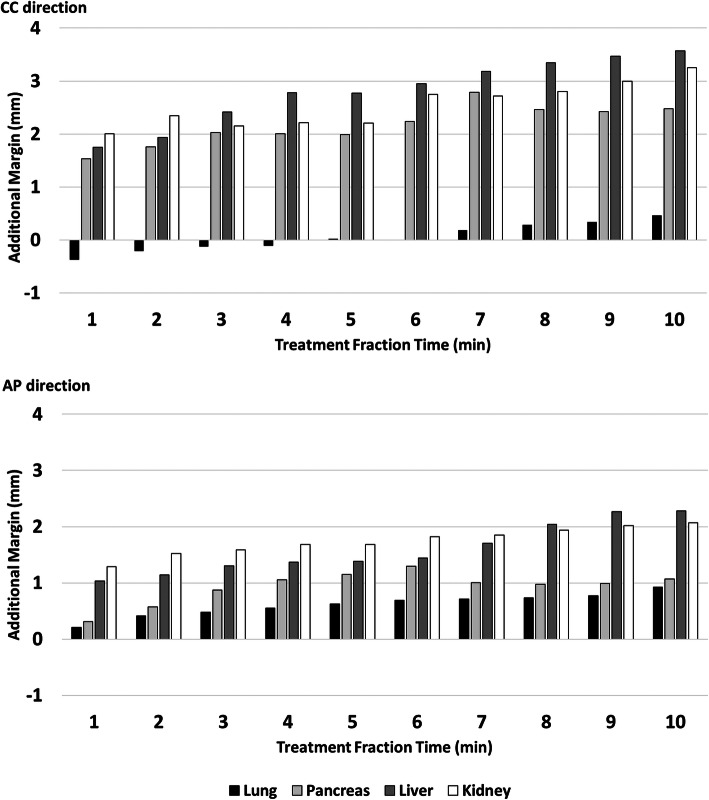
Additional ITV margin to ensure that each lesion is inside the ITV for 95% of treatment time in function of the treatment fraction time. In black the lung cases, in light grey the pancreatic lesions, in dark grey the liver lesions and in white the kidney lesions

Figure 4 reports the additional margin required to make the ITV strategy strongly reliable (%TT ≥ 95%) in CC (upper) and AP (lower) direction. Negative values show that the ITV margins estimated during 4DCT simulation are larger than the effective motion observed during treatment using cine MRI.

Based on these considerations, no additional margin has to be added in case of lung lesions, independently from the TFT (all margins are < 1 mm).

An additional 2 mm margin in CC and 1 mm in AP direction is recommended to make the ITV approach strongly reliable, if the TFT remains ≤4 min, for pancreatic lesions.

With the same TFT (<= 4 min) additional margins of 3 mm in CC and 2 mm in AP are recommended to obtain %TT > 95% in hepatic and renal lesions.

## Discussion

Although the ITV strategy is widely diffused to manage tumour motion in clinical practice, its robustness is still a matter of discussion, with controversial results reported in literature [12, 37, 38].

The findings of this study, taking into account a limited cohort of patients, indicate that the reliability of the ITV approach depends on the considered treatment site, being the result of the interplay of two main factors: the accuracy in the ITV margin definition from 4DCT and the tumour motion variability occurring during the radiation treatment.

An incorrect estimation of the tumour motion amplitude on 4DCT may introduce a systematic error in the ITV margins definition, limiting the accuracy of this strategy regardless of the TFT Several recent experiences observed that the 4DCT imaging can under or over-estimate the tumour motion by more than 3 mm, depending on the lesion location and the extent of the motion amplitude. Furthermore, assuming as appropriate the ITV margin estimated during 4DCT simulation, long- and short-term tumour motion variability during the course of therapy can cause significant displacements of the tumour position, making the initially estimated ITV no longer adequate to cover the lesion trajectory [20, 39].

The results reported in Table 2 show that, for the analysed lung cases, the ITV strategy ensured an appropriate target coverage in CC and AP direction , when the TFT does not exceed 7 min.

In all the observed lung cases, the 4DCT image allowed a correct estimation of the ITV margins , as described in Fig. 3, where all the lung cases present a %TT value higher than 95% in the first minute of treatment, when intra-fraction variability can be considered negligible.

Increasing the TFT, the %TT mainly decreased for lesions with larger motion amplitudes, as demonstrated by the results of the Spearman’s test reported in Table 3: the correlation starts to be significant in CC direction when TFT exceeds 4 min.

The results observed in this study are in line with those published by Britton et al., who observed on 10 lung cases an inferior motion variability in lesions with initial amplitude inferior to 5 mm compared to those observed in lesions with larger initial amplitude [40].

Dhont et al. also observed important variations in motion amplitude between those obtained from 4DCT at simulation and those measured during treatment, for amplitudes above 7 mm [39].

With regard to abdominal lesions, a %TT < 95% was observed at the first minute of treatment in 50% of cases, suggesting that the ITV definition is more challenging in the abdominal site.

In many of these cases, in fact, the target delineation accuracy on different breathing phases resulted to be limited by the low soft-tissue contrast provided by 4DCT and by the presence of blurring effects.

This last aspect can have a higher impact in case of kidney and liver lesions, where the hypodense tissue and the overlap with nearby structures with similar image contrast further limit the quality of the delineation.

Because of these difficulties, the reliability of the ITV approach seems to be lower in the abdominal region compared to the thorax. In order to safely adopt the ITV strategy, the use of extra margins to be added to those estimated by 4DCT is recommended,

especially when no contrast agent is used .

The extent of these additional margins is dependent on TFT, as shown in Fig. 4. This is likely due to the fact that an increase in treatment time causes patient exhaustion, which leads to millimetric displacements of the target and to the manifestation of effects such as baseline drift and shifts, as already demonstrated by previous analysis [20, 22, 37–39].

One of the major limitations of this study is due to the fact that no evaluation can be made in left-right (LR) direction, since all the analyses were carried out on MR images acquired on a single-slice sagittal plane.

Although previous studies showed that LR represents the less significant motion direction (with values of 1–2 mm), a comprehensive analysis in this direction is necessary to express definitive considerations on ITV reliability [6, 39].

It should also be taken into account that an additional set-up margin has to be considered for PTV generation, as recommended by several ICRU reports [41, 42].

The combination of ITV and set-up margin can lead to large planning target volumes, limiting the prescription dose in some cases: for these reasons, active motion management and breath hold techniques should be preferred whenever possible, especially in case of tumours with large motion amplitude.

## Conclusion

This study has evaluated the reliability of the ITV approach in the abdominal and thoracic sites in a cohort of 27 patients undergoing MRgRT.

On the basis of the analysed cases, this approach appears to be reliable in the thorax, where the ITV margins estimated in 4DCT ensures an appropriate target coverage if the TFT does not exceed 7 min.

On the other hand the ITV strategy results more challenging in the abdomen, due to the higher uncertainties in ITV definition and to the larger inter-fraction motion variability observed in the analysed patients.

The addition of extra margins based on the TFT may represent a valid tool to compensate such limitations.

Further studies including larger cohorts of patients and the acquisition of orthogonal planes for motion estimation are recommended to verify the results of this study and achieve more comprehensive evaluation of the reliability of the ITV approach.

## Data Availability

The datasets analysed during the current study are available from the corresponding author on reasonable request.

## Abbreviations

AP: Antero-posterior
CC: Cranio caudal
CT: Computed tomography
ITV: Internal Target Volume
MIP: Maximum Intensity Projection
MR: Magnetic resonance
MRgRT: Magnetic resonance-guided radiotherapy
PTV: Planning target volume
RT: Radiotherapy
SBRT: Stereotactic body radiation therapy
Sec: Seconds
TFT: Treatment fraction time
TLD: Tracking-learning-detection
TrueFISP: True fast imaging with steady state precession
TT: Treatment time
4DCT: Four dimensional computed tomography
4DRT: Four dimensional radiotherapy
